# Comparison of PACS and Bone Ninja mobile application for assessment of lower extremity limb length discrepancy and alignment

**DOI:** 10.1007/s11832-016-0761-5

**Published:** 2016-07-22

**Authors:** Amanda T. Whitaker, Martin G. Gesheff, Julio J. Jauregui, John E. Herzenberg

**Affiliations:** 1 Department of Orthopaedic Surgery, Nationwide Children’s Hospital, 700 Children’s Drive, Columbus, OH 43205 USA; 2 Department of Orthopaedic Surgery, University of California San Francisco, 500 Parnassus Ave, San Francisco, CA 94143 USA; 3 International Center for Limb Lengthening, Rubin Institute for Advanced Orthopedics, Sinai Hospital of Baltimore, 2401 W. Belvedere Ave, Baltimore, MD 21215 USA

**Keywords:** Limb deformity, Bone Ninja, Deformity measurements, Limb length, PACS

## Abstract

**Purpose:**

There are over 500 medically related applications (apps) for mobile devices. Very few of these applications undergo testing and peer-review for accuracy. The purpose of this study is to assess the accuracy of limb deformity measurements on the Bone Ninja app compared to PACS and to determine the intra- and inter-observer variability among different orthopaedic practitioners.

**Methods:**

Four participants (attending, senior and junior resident, and physician assistant) measured the leg length (LL), the lateral distal femoral angle (LDFA), and the medial proximal tibial angle (MPTA) of 48 limbs (24 patients), twice with both Bone Ninja and PACS. The difference between the measurements obtained with the Bone Ninja app and PACS were measured. We determined the consistency of the intra-observer intra-class correlation coefficient (ICC) for both systems.

**Results:**

There were no statistical differences in leg length discrepancy (LLD), MPTA, or LDFA measurements between Bone Ninja and PACS (*p* = 0.96, 0.87, and 0.97, respectively). The intra-observer ICC for the LL, LDFA, and MPTA was similar between Bone Ninja and PACS (0.83, 0.89, and 0.96 vs. 0.96, 0.93, and 0.95, respectively). The inter-observer ICC was similar between Bone Ninja and PACS (0.95, 0.96, and 0.99 vs. 0.99, 0.98, and 0.98, respectively).

**Conclusions:**

This study demonstrates that Bone Ninja is an accurate educational tool for measuring LLD, LDFA, and MPTA. Both systems are reliable instruments for evaluating limb length differences and angles on standing radiographs for pre-operative deformity planning and education. This is the first study to evaluate the accuracy of Bone Ninja compared to the gold standard of PACS.

## Introduction

The reliability of proper pre-operative planning and measurement of lower extremity deformity is important in the treatment of growth disturbances, malalignment, malunions, and unicompartmental arthritis in the young person [1]. Pre-operative planning is typically done with various Picture Archiving and Communication Systems (PACS) and software programs. There has been a flood of novel portable medical applications and an increase in tablet ownership from 64 % in 2011 to 93 % in 2014 [2, 3]. Bone Ninja, a mobile application, was developed for patient/physician education and is available for the iPad platform (Apple Inc., Cupertino, CA, USA). This application allows for measurements and deformity correction planning without the need for scissors, paper, or expensive computer software programs. There are currently over 500 medical applications on the iTunes website for the iPad. Some of these applications assist with medical diagnosis, treatment suggestions, or function as tools using the in-device accelerometer [4, 5]. Very few of these applications have undergone testing and peer-review for their accuracy [6, 7].

Previous studies have compared the gold standard of hard-copy radiographs to PACS measurements for limb deformity evaluation and found the two systems are equal in intra- and inter-observer reliability [8–11]. The scanogram has been compared to the standing antero-posterior (AP) entire leg radiograph and the two have been equivalent in determining leg length discrepancy. However, a standing AP entire leg radiograph can better assess the overall alignment and deformity [9]. Newer computer programs such as TraumaCAD (Brainlab, Westchester, IL, USA) have increased the utility of PACS and have high reliability, but they are often expensive and may not be readily accessible [1]. Bone Ninja is a less expensive alternative to deformity planning using mobile technology and forgoing paper and scissors.

The Bone Ninja planning mobile application has not been evaluated and compared to the gold standard of PACS. Therefore, the purpose of this study is to determine the inter- and intra-observer reliability for measuring limb deformity measurements using Bone Ninja compared to the gold-standard PACS.

## Materials and methods

This study was approved by the hospital Institutional Review Board prior to its initiation. Twenty-four consecutive skeletally-mature patients who were evaluated at the Rubin Institute for Advanced Orthopaedics with full standing alignment films were identified retrospectively. To be included, all lower extremity joints (hip, knee and ankle) had to be visible, with the patella facing forward. Those with joint fusion, external fixator, inadequate patellar positioning, or inadequate stitching were excluded. The films were de-identified, numbered, and randomized four times (twice for PACS and twice as JPEGs for Bone Ninja) through a random number generator. To simplify and standardize the randomization process, image JPEGs were standardized to 666 × 977 pixels at a resolution of 72 dpi.

**Fig. 1:**
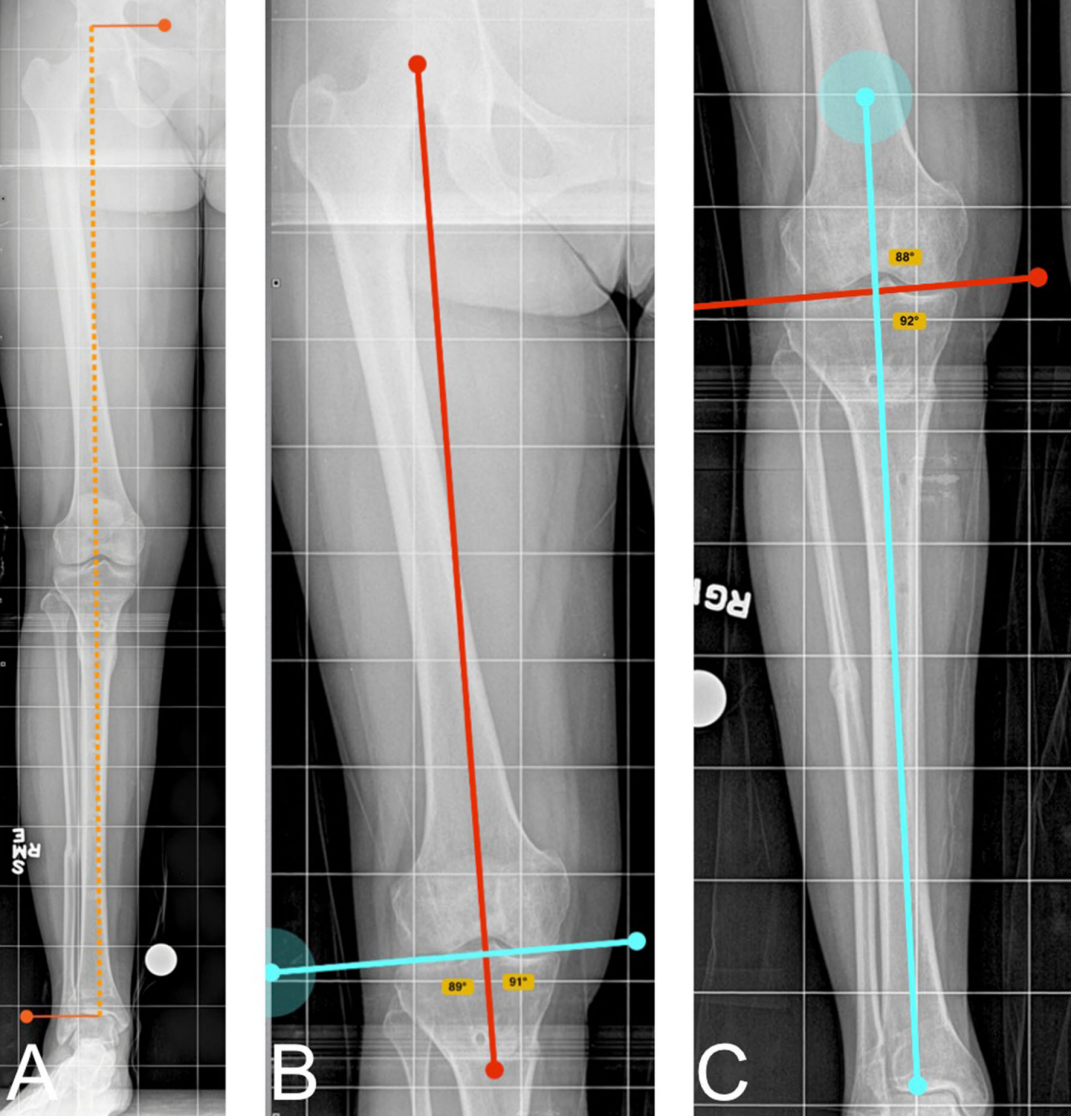
Radiographic measurements as demonstrated on Bone Ninja. **a** Total limb length. **b** Lateral distal femoral angle (LDFA). **c** Medial proximal tibial angle (MTPA)

Four raters, an attending orthopaedic surgeon, a senior orthopaedic resident, a junior orthopaedic resident, and an orthopaedic physician assistant, measured each PACS and Bone Ninja image on four separate occasions with a minimum of 1 week between measurements and alternating PACS and Bone Ninja. They were timed during their last session with Bone Ninja and PACS. Satisfaction was determined by the question of which they would prefer using for deformity measurements. No participant had any financial or developmental involvement in the Bone Ninja app. The PACS measurements (Philips iSite Enterprise PACS, MA, USA) were completed on a 10.5 × 13 inch monitor. The iPad was an iPad 4th generation with 9.7-inch retina display. The four raters were allowed to keep the iPad at the end of the study. The measurements recorded for each image were the right and left total limb length (LL), lateral distal femoral angle (LDFA), and medial proximal tibial angle (MPTA) after calibration of each image using a 2.54-cm calibration ball (Fig. 1). Each participant was given instructions both verbally and on paper, and a hands-on demonstration on how to do the measurements on both Bone Ninja and PACS. The LDFA was measured as the lateral angle between the mechanical axis of the femur, from the center of the femoral head to the center of the knee, and the distal femoral joint line. The MPTA was measured as the medial angle between the mechanical axis of the tibia, from the center of the knee to the center of the tibial plafond, and the proximal tibial joint line.

All data were incorporated into an electronic spreadsheet (Microsoft Excel, Microsoft Office, Redmond, WA, USA). Intra-class correlation coefficients were calculated for the intra-observer and inter-observer LLD, LDFA, and MPTA measurements within both the PACS and Bone Ninja. We determined agreement by a Cohen kappa value: poor if <0.2, fair if 0.21–0.40, moderate if 0.41–0.60, substantial if 0.61–0.80 and good if >0.80 [12].

### Source of funding

This study was partially funded by a research grant provided by the Save-a Limb Fund.

## Results

The intra-observer correlation coefficients were similar and highly correlated for LLD, LDFA and MPTA (Table 1). The limb length kappa value was 0.96 on the PACS and 0.95 on Bone Ninja, signifying an excellent agreement within the four subjects using both methods of limb deformity measurement (Fig. 1a). The LDFA kappa coefficient was 0.93 on PACS and 0.89 on Bone Ninja, signifying an excellent agreement when measuring LDFA within the four subjects (Fig. 1b). The MPTA kappa coefficient had a high correlation of 0.95 on PACS and 0.96 on Bone Ninja, demonstrating agreement between PACS and Bone Ninja for proximal tibial angle measurements (Fig. 1c). All intra-observer correlations were excellent; therefore Bone Ninja limb deformity measurements taken by one observer are as accurate as the measurements on PACS.

**Table 1:** Intra-observer correlation of limb deformity measurements

System	Measurement	Cohen’s kappa value (*κ*)	95 % Confidence interval
Intra-observer intra-class correlation coefficient (ICC)			
PACS	Limb length	0.96	0.95–0.97
	LDFA	0.93	0.91–0.95
	MPTA	0.95	0.93–0.96
Bone Ninja	Limb length	0.95	0.93–0.96
	LDFA	0.89	0.85–0.92
	MPTA	0.96	0.95–0.97

*PACS* picture archiving and communication system, *LDFA* lateral distal femoral angle, *MPTA* medial proximal tibial angle

The inter-observer correlation coefficients, or the agreement among our four participants of all different levels of training, were similar and highly correlated for LLD, LDFA, and MPTA (Table 2). Limb length kappa coefficient was 0.99 for the PACS and 0.98 for Bone Ninja (Fig. 1a). LDFA kappa coefficient was 0.98 for PACS and 0.96 for Bone Ninja (Fig. 1b). MPTA kappa coefficient was 0.98 for PACS and 0.99 for Bone Ninja (Fig. 1c). All of these measurements signify excellent agreement between the observers for both PACS and Bone Ninja.

**Table 2:** Inter-observer correlation of limb deformity measurements

System	Measurement	Cohen’s kappa value (*κ*)	95 % Confidence interval
Inter-observer intra-class correlation coefficient (ICC)			
PACS	Limb length	0.99	0.99–0.99
	LDFA	0.98	0.97–0.99
	MPTA	0.98	0.97–0.99
Bone Ninja	Limb length	0.98	0.97–0.99
	LDFA	0.96	0.94–0.98
	MPTA	0.99	0.98–0.99

*PACS* picture archiving and communication system, *LDFA* lateral distal femoral angle, *MPTA* medial proximal tibial angle

When assessing the LLD, there were no statistical differences between the Bone Ninja and the PACS (*p* = 0.96, 0.87, and 0.97). The right and left leg length measurements were compared for accuracy, and there was no difference in measurement accuracy between right and left on either PACS or Bone Ninja (*p* = 0.526).

The subjects found the Bone Ninja system more enjoyable and faster than PACS (3 min and 43 s per image on Bone Ninja vs 4 min and 51 s for PACS).

## Discussion

There is a call for review of medical apps’ accuracy of content and for peer review [13]. Currently, there are a few websites that review medical apps for their use and the variety of apps available [14]. Very few have addressed the accuracy of their content [7]. This study demonstrates that Bone Ninja is an accurate application for measuring lower extremity deformity, specifically the LLD, LDFA, and MPTA. When comparing Bone Ninja to PACS, the length measurements are slightly more consistent with the PACS. However, the angular measurements are slightly more consistent with the Bone Ninja application. Training level did not affect the accuracy of measurements. Bone Ninja was faster and more enjoyable for the participant. Both systems appear to be excellent instruments to utilize for education and pre-operative planning of lower extremity deformity on standing radiographs. One key factor in accurate measurements is having a known size marker on the radiographs for limb length accuracy.

Previous studies have looked at the accuracy of deformity measurements between hard-copy radiographs and PACS. Khakharia et al. measured LLD, LDFA and MPTA between two observers using hard-copy radiographs and PACS and determined that use of the digital radiographs was as reliable as the hard-copy radiographs [8]. Marx et al. measured mechanical axis, width of tibial plateau and distance from the mechanical axis to the medial tibial plateau between orthopaedic surgeons and radiologists using PACS and hard copies. They found that PACS had a better inter- and intra-observer reliability and was easier to use than hard copies [10]. Our study had better accuracy for measuring LDFA and MPTA using Bone Ninja compared with PACS than these previous studies, suggesting that Bone Ninja and PACS are more similar than PACS and hard-copy radiographs.

There were some limitations of this study. The relatively small size of our cohorts may have given underpowered findings, but due to the high intra- and inter-observer agreement between both cohorts, our conclusions are well supported. Since the use of Bone Ninja is new when compared to current PACS, medical personnel could have had difficulties in using this new platform; however, the time required for measurements with Bone Ninja was reduced compared to PACS. Hence, once medical personnel are more comfortable with this application, the time difference can be magnified. The screen size is larger for PACS than for the iPAD, although measurements were as accurate on the smaller iPad screen as the larger PACS screen. Lastly, this study was performed in a university center, with an ACGME certified orthopedic residency program, hence the reproducibility of our findings may be reduced in tertiary practices.

The Bone Ninja application can be used to measure leg lengths, mechanical and anatomical limb axes, make virtual osteotomies and the apex of the deformity and rotate the segment to correct the deformity. Traditionally, limb deformity planning education has relied on printed radiographs, colored pencils, and scissors. Pictures of radiographs can be taken with the built-in camera on the iPad or uploaded from email by a click on the “add photo” button. In addition, pictures of limbs can be taken by the iPad and corrected through virtual osteotomies. There are hardware and devices available for modeling, such as an external fixator, blade plate, and straight plates. While the measurements can be obtained using the PACS system, the virtual osteotomies, deformity correction, and ease of portability cannot. There are software packages available to make osteotomies and deformity correction, but they are more expensive and less portable than the iPad app Bone Ninja.

This is the first study to evaluate the consistency and reliability of the planning mobile application when compared to the gold standard of PACS. Bone Ninja is as accurate as PACS in measuring and assessing lower extremity deformity, has improved user satisfaction, and is faster. In addition, Bone Ninja allows for in-app osteotomies and correction, negating the need for printing the digital films on paper to cut the osteotomy. This mobile technology allows for reliable, portable pre-operative planning and educational tools that hopefully lead to smoother, more accurate surgeries for complex deformities by better understanding the underlying deformity. It was not our purpose to eliminate PACS planning, nor do we conclude that Bone Ninja is better; however, we believe that Bone Ninja could be considered a powerful tool in the medical armamentarium for limb lengthening and deformity correction, especially when it comes to patient and physician education.
